# Antitumor, antioxidant, and antibiofilm activities of chitooligosaccharides by chitosanase from *Bacillus cereus*

**DOI:** 10.1038/s41598-026-67916-w

**Published:** 2026-09-03

**Authors:** Akram N. Salah, Nooran S. Elleboudy, Mohamed M.S. Farag, Talat A. EL-kersh, Mahmoud A. Yassien

**Affiliations:** 1 Microbiology and Immunology Department, Faculty of Pharmacy, Badr University in Cairo (BUC), Badr City, Cairo, 11829 Egypt; 2 Department of Microbiology and Immunology, Faculty of Pharmacy, Ain Shams University, Organization of African Unity St., Cairo, POB: 11566 Abbassia Egypt; 3 Botany and Microbiology Department, Faculty of Science, Al-Azhar University, Cairo, 11884 Egypt; 4 Biomedical Research Department, Armed Forces College of Medicine (AFCM), Cairo, Egypt; 5 The Regional Center for Mycology and Biotechnology, Al-Azhar University, Cairo, Egypt

**Keywords:** Chitooligosaccharides, Chitosanase - *Bacillus cereus*, Antioxidant, Antitumor- antibiofilm, Biochemistry, Biotechnology, Microbiology

## Abstract

Chitooligosaccharides (COSs) are hydrophilic short oligomers produced by the action of microbial chitosanases on chitosan. The aim of this study is to assess the antitumor, antioxidant, and antibiofilm activities of bio-produced COSs from *B. cereus* strain isolated from Egyptian soil. To evaluate the cytotoxicity of COSs, an MTT assay was performed. To find that COSs exhibit antitumor activity against MCF-7, HepG-2, A549, and CACO-2 cell lines with mean IC_50_ of 818.8 µg/mL, 1845.9 µg/mL, 1293.9 µg/mL, and 1432.9 µg/mL, respectively. In addition, the antioxidant activity of COSs was measured by assessing their 2,2-diphenyl-1-picrylhydrazyl (DPPH) radical-scavenging activity relative to ascorbic acid. The tested COSs showed strong DPPH radical-scavenging activity at high concentration (98.6 ± 0.10%). Assessing the antioxidant activity of COSs was also carried out using the HFD-mice model by measuring catalase and other enzyme levels in blood, stomach, and liver samples. The 0.5% COS dose increased catalase, GSH, and GPx levels in liver, stomach, and serum specimens (*P* < 0.05). The antibiofilm activity of the tested COSs was assayed using a microtiter plate assay against *P. aeruginosa* and *S. aureus* biofilm-forming microorganisms. The tested COS at 125 µg/mL showed promising activity against biofilm formation by *S. aureus* and *P. aeruginosa*, with 51.7 ± 4.2 and 43 ± 2.1, respectively. In conclusion, COSs showed promising antitumor, antibiofilm, and antioxidant activities.

## Introduction

Chitooligosaccharides (COSs) are hydrophilic short oligomers that are produced from the chitosanase hydrolytic action on chitosan polymer^1^. Chitosanase is an enzyme found in bacterial strains such as *Bacillus* sp., *Pseudomona*s sp., and *Streptomyce*s sp^2^. that can selectively hydrolyze the glucosidic bond between the D-glucosamines to produce the bioactive COSs^3^ which are low–molecular-weight derivatives of chitosan composed of 3–11 glucosamine and N-acetylglucosamine units linked through β-(1→4)-O-glycosidic bonds. As oligomeric forms of chitosan, COSs retain many of their biological properties, including antioxidant, antimicrobial, anticancer, immunostimulatory, and anti-inflammatory activities, as well as potential effects against HIV and Alzheimer’s disease^4^.

These bio-produced COSs have a wide range of molecular weights, degrees of polymerization (DP), and degrees of deacetylation (DD)^5^. This enables them to have greater water solubility without the need for pH adjustment, greater bioavailability, and more powerful biological activity^6^.

Interestingly, COSs have various biological activities in medical fields, including anti-tumor activity against different types of cancer^7^, These positively charged polysaccharides have demonstrated the ability to induce apoptosis in a wide range of cancer cells, including those associated with liver, breast, cervical, kidney, and lung cancers, as well as leukemia and colorectal malignancies^8^.

Because oxidative stress is a major public health concern arising from the accumulation of reactive oxygen and nitrogen species, there is increasing interest in identifying novel and safe antioxidants such as COSs that can neutralize the 2,2-diphenyl-1-picrylhydrazyl (DPPH) radical scavenging activities or enhancing the serum or organ levels of some antioxidant enzymes such as catalase, superoxide dismutase enzymes, or glutathione by blocking the free radical chain reactions^9,10^.

COSs also exhibit potent antibiofilm activity through multiple mechanisms. Their cationic nature allows strong electrostatic interactions with negatively charged microbial cell surfaces, leading to membrane disruption and increased permeability. In addition, COSs can interfere with the initial adhesion phase of biofilm formation, preventing microbial attachment to surfaces^11–13^.

Despite the well-documented safety profiles of COSs and the promising biological activities demonstrated in various in vitro and in vivo studies, further investigation is still required to fully elucidate their mechanisms of action and therapeutic potential. Therefore, the present study was conducted to explore and evaluate some of COSs’ biological activities, providing deeper insights into their potential application as alternative or adjunct agents. The present study used the purified COSs produced by *Bacillus cereus* PP093334.1 to investigate the antitumor, antioxidant, and antibiofilm activities.

## Methods

### Microorganism and production of COSs

The *Bacillus cereus* strain PP093334.1 was obtained from Egyptian soil and identified by 16 S rRNA gene sequencing. The COS preparation used in the present study was obtained from the same purified and lyophilized production batch previously described in our study^14^. Briefly, the COSs mixture was bio-produced via enzymatic hydrolysis of colloidal chitosan by chitosanase secreted by the locally isolated *Bacillus cereus* strain SE42. A 5-mL bacterial inoculum (1.5 × 10⁸ CFU/mL) was added to 100 mL of chitosanase-detecting medium containing 1% colloidal chitosan, and the mixture was incubated at 30 °C for 48 h with shaking at 120 rpm. After fermentation, bacterial cells were removed by centrifugation at 6,500 × *g* and 4 °C for 20 min. COSs were purified using Sephadex LH-20 size-exclusion chromatography and subsequently characterized using an ACQUITY UPLC system equipped with a BEH C18 column (1.7 μm, 2.1 × 50 mm) coupled to an electrospray-ionization mass spectrometer, followed by freeze-drying for storage and subsequent experiments. The process yielded 2.16 g of COSs, with a maximum chitosanase activity of 1,190 ± 20.1 U/L. The chromatographic and mass-spectrometric profiles were used to determine the molecular mass distribution and degrees of polymerization of the COS components, with degrees of polymerization (DP1-DP6) and molecular weight ranges determined by mass-to-charge ratio (m/z) from 160.7 to 993.6896 m/z^14^.

### Cytotoxicity

#### Cytotoxic effect on tumor cells using the MTT assay

At first, the safety of the COSs mixture was assessed by testing a wide range of concentrations (50–8000 µg/mL) of COSs against Vero 2 cells.

The tumor cell lines include MCF-7, HepG2, A549, and CACO-2 for breast cancer, hepatocellular carcinoma, lung cancer, and colorectal adenocarcinoma, respectively. They were obtained from the Regional Center for Mycology & Biotechnology at Al-Azhar University, Cairo, Egypt. The culture medium was Dulbecco’s Modified Eagle Medium (DMEM) (Sigma-Aldrich, St. Louis, USA), and fetal bovine serum (FBS) (10% w/v) (Lonza Co., Belgium) was added to the medium, along with streptomycin/penicillin (1% w/v). Cell lines were cultured in a humidified CO_2_ (5%) incubator at 37 ℃. The growth medium was replenished every 2 to 3 days^15^. The viable cells were determined by the (3-[4,5-dimethylthiazol-2-yl]-2,5-diphenyltetrazolium bromide (MTT) test as previously described by Mosmann^16^ and modified by Dias et al.^17^. After 24, 48, and 72 h of incubation, cell viability (%) was measured.

The 50% inhibitory concentration (IC_50_) is the concentration of a tested COSs that inhibits or is toxic to about 50% of cells^18^. The cytotoxicity % was calculated as follows:

$$ {\mathrm{Cell}}~{\mathrm{growth}}~{\mathrm{inhibition}}~\left( \% \right){\text{ }} = {\text{ 1}}00{\text{ }} - {\text{ COSs}} - {\text{treated cells absorbance}}/{\text{control cells absorbance }} \times {\text{ 1}}00 $$

#### Microscopic observation of the tumor cells treated with COSs

The cancer cell lines (MCF-7, HepG2, A549, and CACO-2) were treated with COSs at a concentration of 1000 µg/mL, and after 48 h of incubation, cellular morphology was visualized and examined using an inverted microscope (CKX41; Olympus, Japan)^19^. All images were analyzed for morphological changes using ImageJ (Linux, x86-64, US).

### Antioxidant

#### In vitro measurement of antioxidant activity on DPPH

The experiment was conducted as described in the method of Yen and Duh^20^, modified by Yeo et al.^21^. Different concentrations of COSs (ranging from 2 to 1000 µg/mL) in methanol were placed in test tubes (2 mL each) in triplicate. To each test tube, 2 mL of 0.1 mM DPPH dissolved in analytical-grade methanol, protected from light until use, was added and then thoroughly mixed^21^. The reaction tubes were immediately covered with aluminum foil and incubated in the dark at room temperature. After a 30-minute incubation at 27 °C, the tested samples were measured using a UV-Vis spectrophotometer (Milton Roy, Spectronic 1201). Absorbance at 515 nm was continuously monitored, and data were recorded at 1-minute intervals until the absorbance reached a stable state. The absorbance of DPPH radical in the absence of an antioxidant (negative control) and in the presence of ascorbic acid (positive control) was investigated. The IC_50_ values for COSs and ascorbic acid, and the DPPH radical scavenging activity (%) were calculated.

The DPPH radical-scavenging activity was calculated using the following equation:

$$\:\text{DPPH radical-scavenging activity}(\%)=\frac{{A}_{\mathrm{c}\mathrm{o}\mathrm{n}\mathrm{t}\mathrm{r}\mathrm{o}\mathrm{l}}-{A}_{\mathrm{s}\mathrm{a}\mathrm{m}\mathrm{p}\mathrm{l}\mathrm{e}}}{{A}_{\mathrm{c}\mathrm{o}\mathrm{n}\mathrm{t}\mathrm{r}\mathrm{o}\mathrm{l}}}\times\:100$$

where $\:{A}_{\mathrm{c}\mathrm{o}\mathrm{n}\mathrm{t}\mathrm{r}\mathrm{o}\mathrm{l}}$ represents the absorbance of the DPPH solution prepared with the corresponding solvent without the tested sample, and $\:{A}_{\mathrm{s}\mathrm{a}\mathrm{m}\mathrm{p}\mathrm{l}\mathrm{e}}$represents the absorbance of the reaction mixture containing DPPH and the tested sample.

#### In vivo measurement of antioxidant activity of COSs

##### Animals

All female BALB/c mice used in the in vivo experiment in this study were obtained from the Animal House Facility, Faculty of Pharmacy, Ain Shams University, Cairo, Egypt, with the required institutional permission for their use in the study, which was granted by the Faculty of Pharmacy, Ain Shams University’s Ethical Committee (The Research Ethics Committee (REC) approval no. REC-289).

Healthy female BALB/c mice aged 8–11 weeks and weighing 21–39 g at the beginning of the experiment were used. Before experimental allocation, all animals were examined and confirmed to be clinically healthy, with normal activity, appearance, and feeding behavior and no visible signs of disease or injury.

Mice were housed under controlled environmental conditions at 25 ± 2 °C and 40–65% relative humidity, with a 12-h light/12-h dark cycle. The animals were housed at a density of 8 mice per cage in standard polypropylene cages containing autoclaved wood-shaving bedding. A standard laboratory rodent diet and clean drinking water were provided ad libitum throughout the study. The animals were allowed to acclimatize to the housing conditions one week before the experimental procedures.

Acute toxicity assessment was first carried out as reported by our previous study; the safety of the COS preparation was previously evaluated following oral administration to mice at doses of 50, 75, 100, 250, 500, and 1000 mg/kg body weight. Animals were continuously observed for the first 4 h for changes in alertness, spontaneous activity, irritability, salivation, urination, piloerection, and corneal and pinna reflexes. Mortality and delayed signs of toxicity were monitored for 14 days. No mortality, behavioral abnormalities, irritation, or visible toxic effects were detected at any tested dose^14^.

This experiment was carried out as described by Qu and Han^22^, with slight modifications. In brief, four groups of female Balb/c mice (each included 6 mice) were included in this experiment. The groups are described as follows; group I the control group which fed a normal diet (9-10.2% fat content), group II a group of mice which fed a high-fat diet (HFD) (40.8–44.8% fat content), and group III was a group of mice which HFD with purified COSs (0.5%), and group IV was a group of mice which fed HFD with purified ascorbic acid as a positive control (100 mg/kg). This experiment was carried out for six weeks. The mice in this experiment were fasted for 24 h before performing the experiment.

COSs were incorporated into the HFD at a concentration of 0.5% (w/w), equivalent to 5 mg COS/g diet. Assuming an average dietary consumption of approximately 4 g/mouse/day, the estimated daily COSs intake was approximately 20 mg/mouse/day. Based on the mean body weight of the COS-treated group during the experiment, calculated from the initial and final mean body weights * (35.4+41.2)/2=38.3g*This intake corresponded to approximately 522 mg/kg body weight/day. The estimated exposure ranged from approximately 565 mg/kg/day at the beginning of the experiment to 485 mg/kg/day at the end.

Daily observations were conducted on each group of mice. Mice’s body weight was measured before treatment and 6 weeks after treatment. After 6 weeks of HFD intake, blood samples were collected from each group of mice and centrifuged at 4,000 g for 10 min at 4 °C to obtain serum samples. The mice were euthanized by cervical dislocation, and their livers and stomachs were excised, rinsed, weighed, and homogenized in suitable physiological saline to obtain a 10% w/v homogenate. The liquid mixture was acquired for further biochemical analysis.

### Biochemical assays

Determination of reduced glutathione (GSH) and glutathione peroxidase (GPx) was carried out as described by Paglia and Valentine^23^. The activities of catalase and superoxide dismutase (SOD) were assessed according to the methods of Beyer Jr. and Fridovich^24^ and Aebi^25^.

### Antibiofilm assay

The biofilm-forming *S. aureus* (ATCC 6538) was obtained from the Faculty of Pharmacy, Ain Shams University. In contrast, *a P. aeruginosa* clinical isolate was previously reported to be biofilm-forming in Elshamy et al.^26^. study. The assessment was conducted in 96-well polystyrene flat-bottom plates as described previously by Ishwarya et al.^27^.

An aliquot of 300 µl of freshly inoculated TSB containing 1% glucose with a final concentration of 10^6^ CFU/mL was placed into each well of a microplate and cultivated in the presence of predetermined sub-inhibitory COSs concentrations.

### Determination of MIC of COSs

The minimum inhibitory concentration (MIC) of the purified COSs was determined by broth microdilution against the tested biofilm-forming microorganisms according to Clinical Laboratory Standards Institute (CLSI) guidelines^28^.

As controls, wells containing medium and those without COSs were utilized. The plates underwent incubation at 37 °C for 48 h. Following incubation, the liquid above the cells was removed, and each well was carefully rinsed with phosphate-buffered saline. The wells were then left to dry in the air for 30 min. The formed biofilm was then stained for 2 min at room temperature with 150 µL of 0.1% crystal violet solution. The excess stain was then removed by rinsing the plate several times with sterile distilled water. To solubilize the dye attached to the cells, 250 µl of 33% acetic acid was added to each well. After 15 min of incubation, absorbance was measured using a microplate reader (BioTek Instruments, Inc., Tigan, United States) at 570 nm. The following equation is used to determine the antibiofilm effect of COS:

$$ {\text{Biofilm inhibition}}\% {\text{ }} = {\text{ OD Control }}{-}{\text{ OD Sample}}/{\text{OD Control}} \times {\mathrm{1}}00 $$

### Statistical analysis

All experiments were performed in triplicate. Statistical analyses were performed using GraphPad Prism version 8.0.1. Data are presented as the mean ± standard deviation. For the cytotoxicity experiments, two-way analysis of variance (ANOVA) was performed separately for each cell line, with COSs concentration and incubation time as the two factors. The interaction between concentration and incubation time was also evaluated. For the DPPH radical-scavenging assay, two-way ANOVA was used to assess the effects of antioxidant treatment (COSs or ascorbic acid), concentration, and their interaction at the concentrations common to both treatments. IC₅₀ values were estimated separately using nonlinear regression of the concentration–response data.

Antibiofilm results were analyzed using two-way ANOVA, with COS concentration and bacterial species (*S. aureus* or *P. aeruginosa*) as the two factors. Body-weight data were analyzed using a two-way repeated-measures ANOVA, with dietary treatment group as the between-subjects factor and time (initial and six weeks) as the within-subjects factor.

The antioxidant biochemical parameters, including catalase, SOD, GSH, and GPx, were analyzed separately for each tissue or serum sample using one-way ANOVA, with dietary treatment group as the independent factor. When a significant overall effect or interaction was detected, Tukey’s multiple-comparisons test was performed. Differences were considered statistically significant at *P* < 0.05.

## Results

**Fig. 1:**
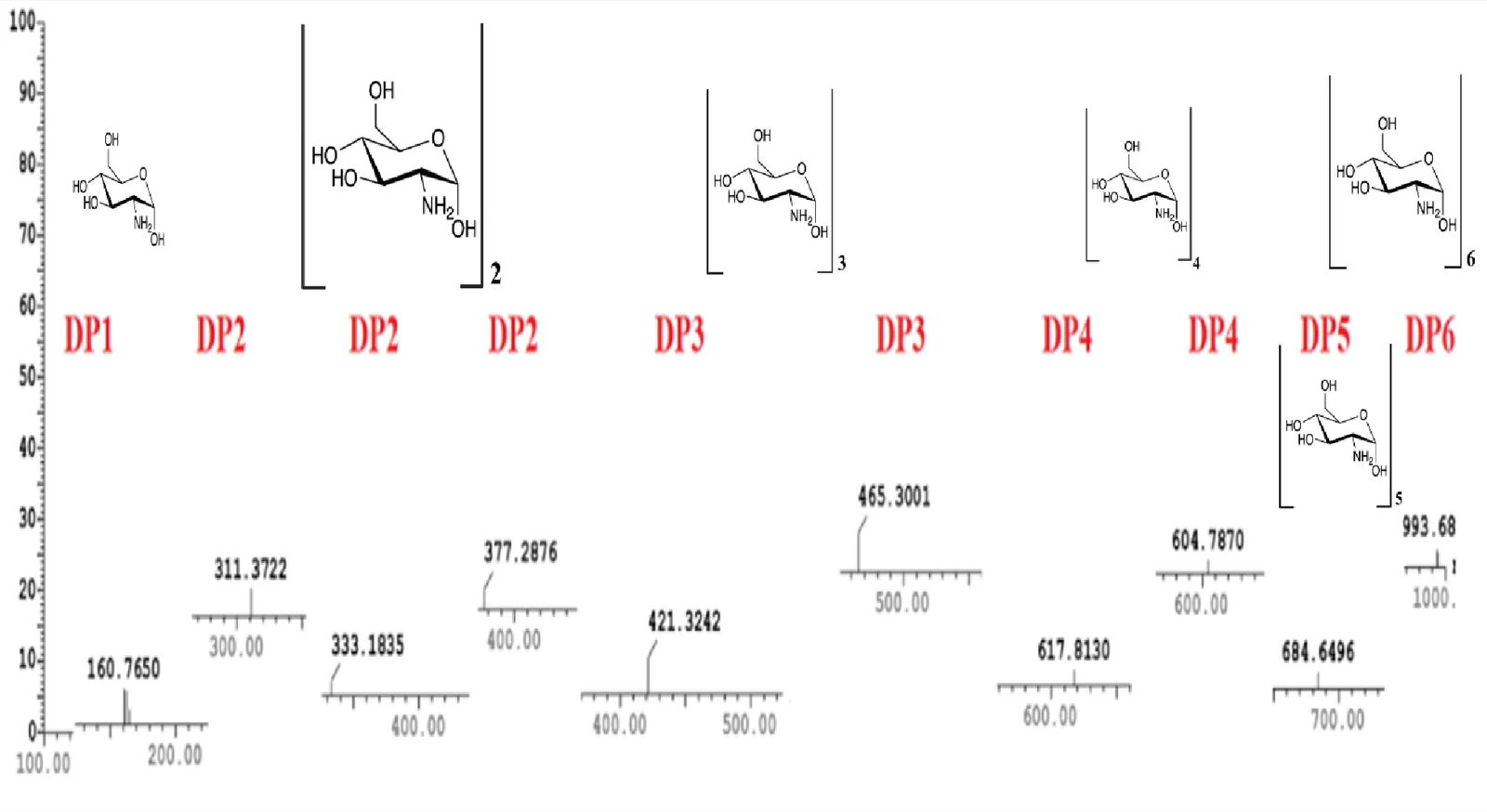
LC mass spectroscopy of COSs mixture obtained from *B. cereus*^14^.

The representative UPLC chromatogram of the COS preparation showed multiple resolved peaks corresponding to oligomers with degrees of polymerization from DP1 to DP6. LC–ESI–MS analysis further detected ions within the m/z range of 160.7–993.6896, supporting the presence of COS components with different molecular masses. These results confirmed the compositional profile of the COS mixture used throughout the present experiments (Fig. 1 ).

**Fig. 2:**
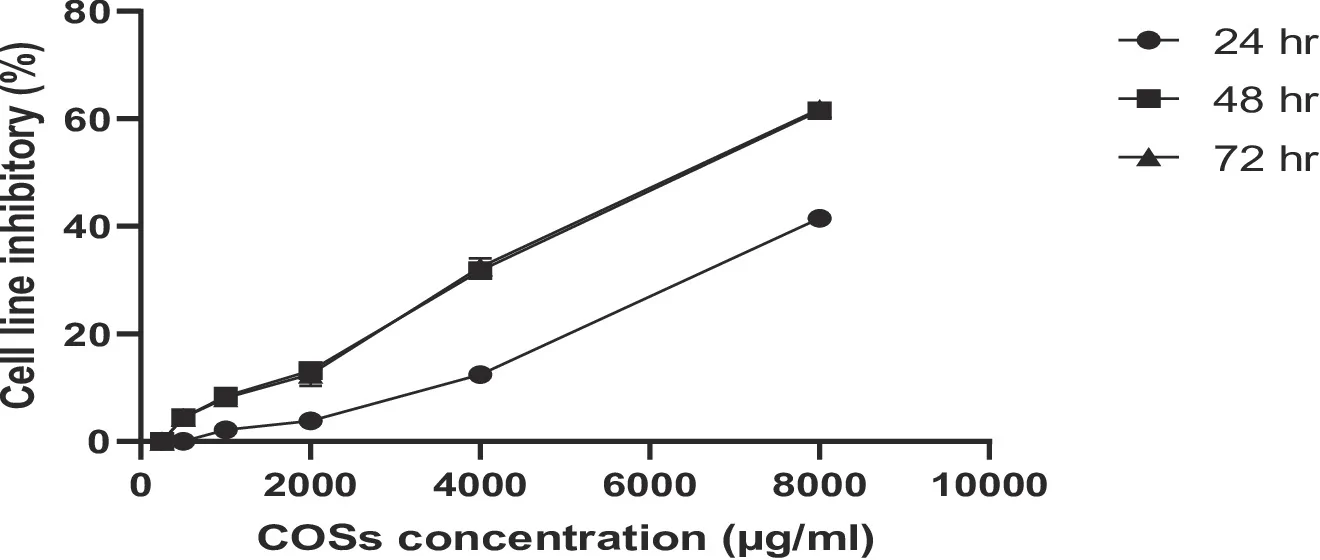
Effect of COSs on cell line inhibitory activity at different incubation times. Cells were exposed to increasing concentrations of COSs (0–8000 µg/mL) and evaluated after 24, 48, and 72 h. Data points represent measured cell-inhibition values at each concentration.

Regarding the cytotoxic effect of COSs on Vero 2 cells, the results showed an IC_50_ of 6410 ± 142.5 µg/mL (Fig. 2).

**Fig. 3:**
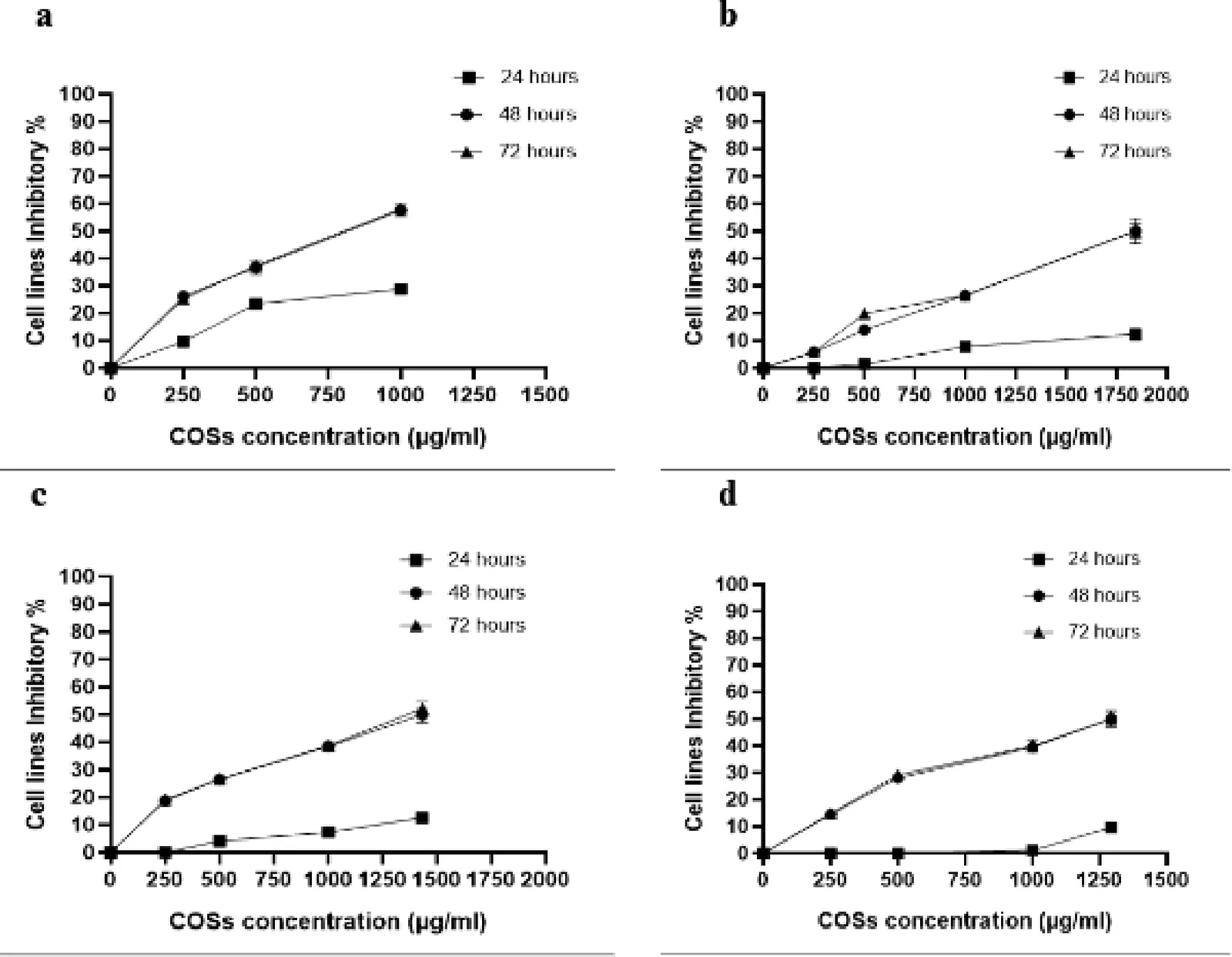
Dose- and time-dependent inhibitory effects of COSs on different cell lines. Cells were treated with increasing concentrations of COSs and assessed at 24, 48, and 72 h. The inhibitory effect of COSs, concentration range 250–1500 µg/ml, on cell line proliferation of (**a**) HepG-2, (**b**) MCF-7, (**c**) A549, and (**d**) CACO-2 cell lines in terms of means ± SD after 24, 48, and 72 h of incubation.

The results showed that the cytotoxic effect of COSs against the cancer cell lines used (MCF-7, A549, HepG-2, and CACO-2) was concentration-dependent. Treatment with 250 µg/mL of COSs caused a reduction in the mean cell line inhibitory percentage to 5.7, 19.1, 25.2, and 14.8% after 72 h incubation periods, while increasing the concentration to 1000 µg/mL caused a reduction in the mean cell line inhibitory percentage to 26.54, 38.7, 37.4, and 39.95%, respectively. Across different incubation periods, increasing the incubation time from 24 h to 48 h was associated with a significant increase in cytotoxicity. In the presence of 1000 µg/mL of COSs, the mean percentage of cell line inhibitory percentage after 24 h was 7.8%, 7.31%, 28.9% and 1.1%, while after 48 h incubation, the percentage of reduction in the number of viable cells was 26.5%, 38.4%, 57.6% and 39.5%, respectively However, no significant difference in the cytotoxic effects of COSs after 48 and 72 h incubation period for all the tested COSs concentrations (Fig. 3). The IC_50_ values of COSs were 818.8 µg/mL, 1845.9 µg/mL, 1293.9 µg/mL, and 1432.9 µg/mL, respectively against MCF-7, HepG-2, A549, and CACO-2 cell lines, respectively after treating with COSs for 48 h.

**Fig. 4:**
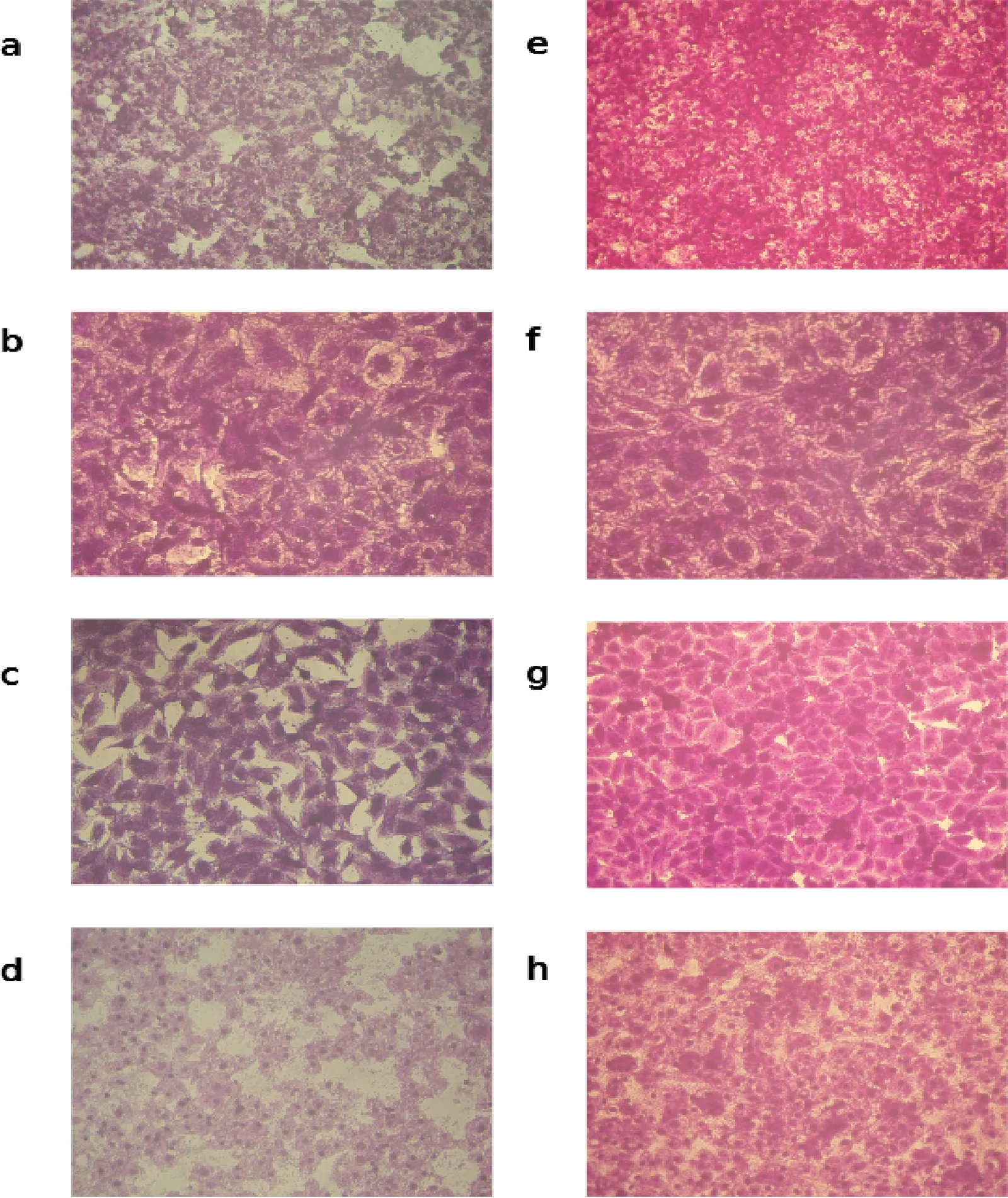
Morphological assessment of cytotoxic effects induced by COSs on cultured cell lines. Representative microscopic images show untreated control cells (right panels) and COSs-treated cells (left panels) at corresponding concentrations and/or exposure times on (**a**) HepG2, (**b**) MCF-7, (**c**) A549, (**d**) CACO-2 cells. While the control includes the untreated cell lines (**e**) HepG2 cells (**f**) MCF-7 (**g**) A549 (**h**) CACO-2 cells.

Microscopic examination of cancer cell lines after treatment with 1000 µg/mL COSs revealed a significant cytopathic effect in A549, HepG-2, and CACO-2 cells. On the other hand, no cytopathic effect was observed in the treated MCF-7 cells. In detail, Control cells exhibit normal morphology, with intact cell membranes, uniform shape, and dense cell-to-cell adherence. In contrast, COSs-treated cells display clear cytotoxic alterations, including cell shrinkage, rounding, detachment from the surface, reduced cell density, and loss of normal cellular architecture. Increased cytoplasmic granulation and disrupted cell integrity are also evident in treated groups. These morphological changes become more pronounced with increasing COSs concentration and/or prolonged exposure, indicating a dose- and time-dependent cytotoxic effect (Fig. 4). Treatment resulted in visible morphological alterations, including cell shrinkage, rounding, loss of adherence, and reduced cell density. These changes were consistent with the cytotoxic effects demonstrated by the cell-viability assay. However, morphological observations alone cannot confirm apoptosis or distinguish it from other forms of cell death.

In the DPPH experiment, the radical-scavenging percentage was measured at different concentrations of COSs (2–1000 µg/ml) and ascorbic acid (0.5–1000 µg/ml) as a positive control.

**Fig. 5:**
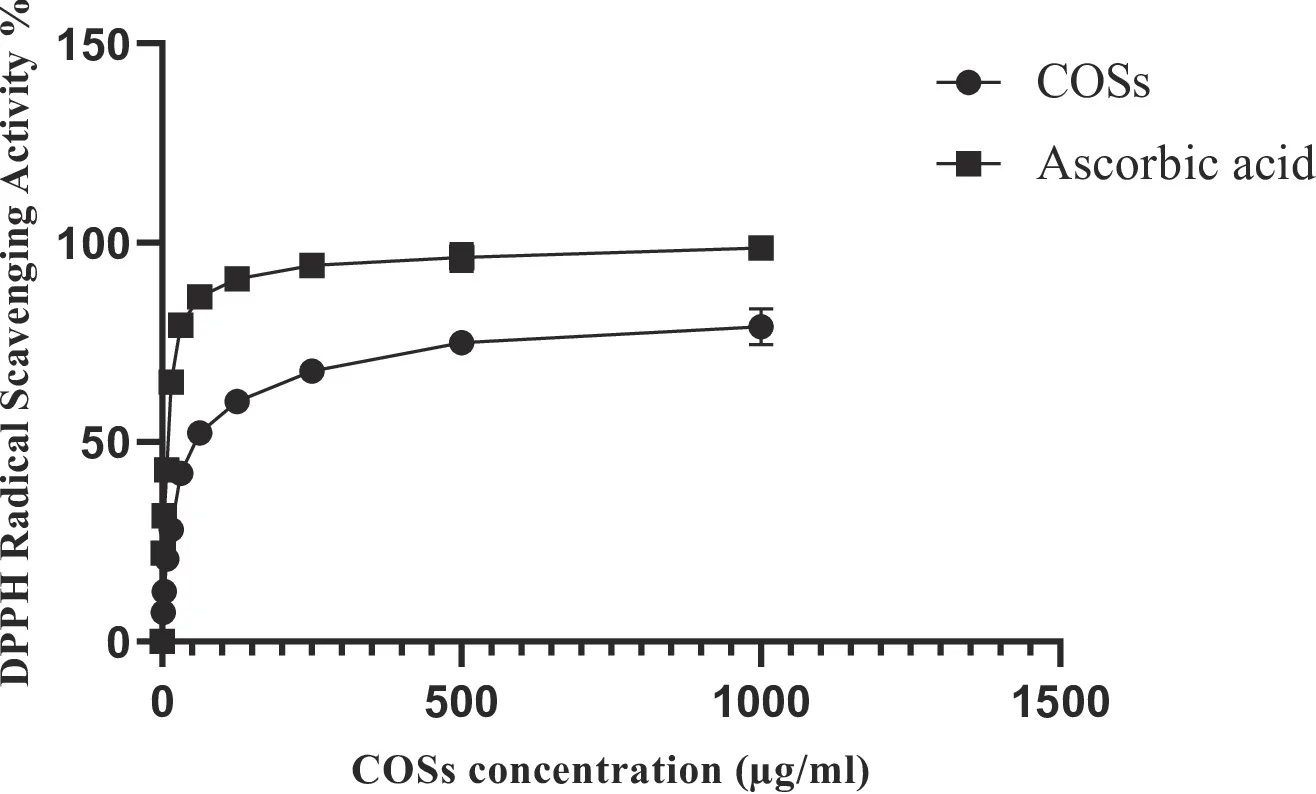
The antioxidant activity (DPPH radical scavenging percentage) of purified COSs and ascorbic acid at concentration ranges (2-1000 and 0.5–1000 µg/mL, respectively).

Along with the tested concentrations of COSs and ascorbic acid, a significant difference (*p* < 0.001) was observed in the DPPH radical scavenging percentage (9.3 ± 0.4% to 98.6 ± 0.1% and 7.3 ± 0.7% to 78.8 ± 0.3%, respectively), with IC_50_ 55.5 ± 1.5 µg/mL and 10.2 ± 0.7 µg/mL, respectively (Fig. 5).

The mice’s body weight after 6 weeks of initiating the experiment showed a significant increase (*P* = 0.0001) in the HFD groups (II, III, and IV). The initial weights of the mice in these groups were 33.4, 35.4, and 32.4 g, and after 6 weeks, the weights increased to 41.4, 41.2, and 38.6 g, respectively. However, there was no significant increase (*P* = 0.387) in group I (the normal diet group, control) (Table 1).

**Table 1:** The change in body weight among the mice studied in this study.

Groups		Initial weight (g)	Weight after 6 weeks (g)
I	Control	29.4 ± 1.74	30.8 ± 3.25
II	HFD	33.4 ± 2.5*	41.4 ± 4.41*
II	HFD + 0.5% COSs	35.4 ± 2.33*	41.2 ± 2.92*
IV	HFD + 1% ascorbic acid	32 ± 1.79*	38.6 ± 2.58*

*It means that there is a significant difference between columns (*P* < 0.05).

The catalase, SOD, GSH, and GPx levels were measured in the collected stomach, liver, and serum specimens.

In stomach tissue, the results demonstrate that the HFD group exhibited a marked reduction in antioxidant enzyme activities, particularly catalase and SOD, compared with the normal group, indicating oxidative stress induction. Conversely, GSH and GPx levels were significantly elevated, suggesting a compensatory response to increased oxidative burden (*P* < 0.01). Administration of COSs (0.5%) significantly improved antioxidant status, as evidenced by increased catalase activity and restoration of GSH levels compared with the HFD group.

In liver tissue, the HFD group showed decreased antioxidant defense, particularly in SOD activity, along with elevated GPx levels, reflecting oxidative stress and hepatic imbalance. Treatment with COSs significantly enhanced antioxidant capacity, increasing catalase, SOD, and GPx activities compared with the HFD group (*P* < 0.01).

In serum, the HFD group showed elevated GPx and catalase levels, indicating systemic oxidative stress. However, antioxidant capacity appeared dysregulated rather than effectively protective. Supplementation with COSs significantly increased catalase, SOD, GSH, and GPx levels compared with the HFD group (*P* < 0.01).

As summarized, compared with the normal diet and HFD groups, there is a significant increase (*P* < 0.0001) in catalase and GSH enzyme levels in all collected specimens. While GPx showed a significant increase (*P* < 0.001) in liver and serum specimens. Regarding the SOD enzyme, a significant increase (*P* < 0.001) in its level was observed in serum specimens only (Table 2).

**Table 2:** The effect of COSs on catalase, SOD, GSH, and GPx in the (a) stomach, (b) liver, and (c) serum.

Group no.	Group name	Catalase (U/g)	SOD (U/g)	GSH (mg/g tissue)	GPx (nMol/g)
a					
I	Normal	8.9 ± 0.1*	405.4 ± 6.8	55.5 ± 1.5*	17.4 ± 0.5*
II	HFD	3.8 ± 0.2	354.3 ± 8.3	86.2 ± 2.3	24.8 ± 1.1
II	HFD + 0.5% COSs	8.5 ± 0.2*	370.2 ± 7.2	93.3 ± 1.5*	21.7 ± 1.1
IV	HFD + 1% ascorbic acid	9.7 ± 0.3*	381.5 ± 5.3*	88.8 ± 1.4*	20.3 ± 0.5
b					
I	Normal	8.7 ± 0.1*	445.7 ± 16.7	57.01 ± 1.8*	21.25 ± 1.3*
II	HFD	8.4 ± 0.2	359.3 ± 14.6	66.24 ± 1.6	33.93 ± 1.2
II	HFD + 0.5% COSs	8.9 ± 0.1*	381.6 ± 9.1	69.95 ± 0.4*	39.29 ± 1.1*
IV	HFD + 1% ascorbic acid	8.4 ± 0.1	533.9 ± 27.1*	56.51 ± 0.5*	24.55 ± 1.1
c					
I	Normal	3.4 ± 0.5*	216 ± 22.5*	4.8 ± 0.5*	38.5 ± 1.6*
II	HFD	4.1 ± 0.6	225.2 ± 22.7	5.1 ± 0.2	52.5 ± 2.3
II	HFD + 0.5% COSs	5.8 ± 0.6*	230.1 ± 7.4*	5.5 ± 0.3*	62.29 ± 2.2*
IV	HFD + 1% ascorbic acid	3.4 ± 0.4	213.2 ± 16.8	5.5 ± 0.2*	55.55 ± 2.2*

SOD: Superoxide dismutase, GSH: Reduced glutathione, and GPx: Glutathione peroxidase.

*It means that there is a significant difference between rows (*p* < 0.01).

The MICs of the purified COSs against *P. aeruginosa* and *S. aureus* were 300 µg/mL and 250 µg/mL, respectively. The antibiofilm assay of sub-inhibitory concentrations of COSs was determined against strong biofilm-forming *P. aeruginosa* and *S. aureus.*

It has been noted that the antibiofilm activity of COSs against the tested isolates was concentration-dependent. Along the tested concentration of COSs (125–31.25 µg/mL), the percentages of decrease in the biofilm formation of *S. aureus* and *P. aeruginosa* were 7.6 ± 0.96–51.7 ± 4.2 and 4.1 ± 1.1–43 ± 2.1%, respectively.

**Fig. 6:**
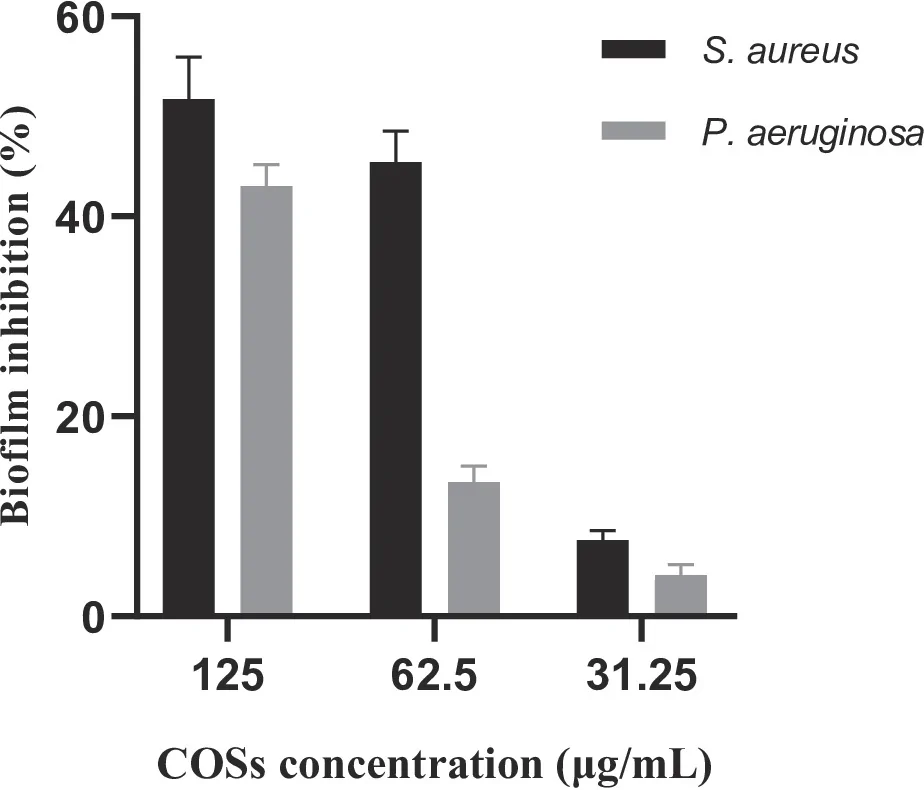
The Antibiofilm of COSs against biofilm-forming *Staphylococcus aureus* and *Pseudomonas aeruginosa* strains.

The tested COS at 125 µg/mL showed promising activity against biofilm formation by *S. aureus* and *P. aeruginosa* strains (Fig. 6).

## Discussion

Chitosan hydrolysis by the enzymatic action of bacterial chitosanase yields chitooligosaccharides (COSs) with different molecular weights and degrees of polymerization, which exhibit distinct biological activities, including antimicrobial, antibiofilm, anti-inflammatory, antioxidant, and antitumor activities ^7, 9^.

In this study, a locally isolated *B. cereus* strain presented a purified COSs mixture containing different degrees of polymerization (DP1-DP6) and a molecular weight range of 160.7. to 993.6896 m/z, which was obtained as previously reported by our previous study ^14^.

The produced COSs showed high safety against normal cells, with an IC_50_ of 6410 ± 142.5 µg/ml. This high IC_50_ value indicates that COSs require very large concentrations to exert toxic effects on normal cells, confirming their excellent biocompatibility and selective safety profile. So, the non-toxic properties of COSs and their being well-tolerated by living tissues are a crucial requirement for COSs’ employment in medicine ^29^.

COSs also exhibited cytotoxicity against HepG-2 and MCF-7 tumor cells, with IC_50_ values of 818.8 µg/mL and 1845.9 µg/mL, respectively. These lower IC_50_ values compared with those in normal cells demonstrate a selective anticancer effect, suggesting that COSs preferentially target malignant cells. A morphological change in HepG-2 cells was observed after 48 h of incubation. Similarly, El-Sayed et al. ^30^. reported that COSs with different molecular weights (300–900 m/z) exhibited cytotoxicity with IC50 values of 1.564 and 2.208 µg/mL against HepG-2 and MCF-7 cells, respectively. In addition, the promising cytotoxic activity of COSs against HepG-2 cells was reported by Shen et al. ^31^. and de Assis et al. ^32^. They reported that the prepared COSs reduced HepG-2 cell viability, with IC_50s_ of 912 µg/mL and 500 µg/mL, respectively.

In addition, the purified COSs in the present study showed promising cytotoxicity against A549 and CACO-2, with IC_50_ values of 1293.9 µg/mL and 1432.9 µg/mL, respectively. A significant morphological change was observed in both cell lines after 48 h of incubation. These structural changes further support the hypothesis that COSs induce apoptosis rather than necrosis, which is preferable for therapeutic applications. This result aligns with Shen et al. ^31^, who reported that COSs inhibited colorectal cancer cell lines (COLO-205) with an IC_50_ of 1000 µg/mL. In addition, the cytotoxic activity of COSs against A549 and another breast cancer cell line, BT-474 (IC_50_ of 500 µg/mL and 3000 µg/mL, respectively), was reported by Ngo et al. ^33^. and Mallakuntla et al. ^34^.

Although the observed cell shrinkage, rounding, detachment, and reduction in cell density may be compatible with apoptosis-associated morphological alterations, the present study did not include specific mechanistic assays to confirm apoptosis. Therefore, the findings are interpreted as evidence of cytotoxicity rather than definitive evidence of apoptotic cell death.

The relatively high IC_50_ values indicate modest direct in vitro cytotoxic potency rather than strong cytotoxic activity. Direct comparison with purified low-molecular-weight anticancer compounds should be made with caution because the present material was tested as a heterogeneous COSs mixture, and its concentration was expressed as the total mass of multiple oligomeric components rather than the molar concentration of a single structurally defined active compound.

The biological activity of COSs is strongly influenced by their molecular weight, degree of polymerization, degree and pattern of acetylation, purity, and chemical substitution. Similarly, Chokradjaroen et al. demonstrated that the cytotoxicity and selectivity of COS preparations varied with their molecular weight distribution. Moreover, El-Sayed et al. reported markedly greater cytotoxicity for fractions within the 10–100 kDa range, whereas their fraction below 1 kDa showed no detectable activity against the examined tumor cells. This difference in molecular-size distribution may partly explain the lower potency of the predominantly low-molecular-mass preparation tested in the present study ^7, 8^.

Despite their modest potency, the tumor-cell IC₅₀ values were lower than the IC₅₀ of 6410 ± 142.5 µg/mL obtained in Vero cells. The results showed preferential cytotoxicity toward malignant cells. Nevertheless, these findings should be considered preliminary evidence of selective, weak-to-moderate in vitro cytotoxicity and not confirmation of therapeutic antitumor efficacy. Fractionation of the mixture by degree of polymerization, detailed characterization of its acetylation pattern, and mechanistic and in vivo antitumor studies are required to identify the most active COS components and to determine their therapeutic relevance.

Purified COSs have antioxidant activity, which is reported by their ability to diminish DPPH radicals ^35^. This study found that the antioxidant activity was in a dose-dependent manner from 2 µg/mL to 1000 µg/mL (*p* < 0.001), and the highest antioxidant activity (78.8 ± 0.3%) for COSs was observed at its maximum concentration (1000 µg/mL) as compared to the positive control, ascorbic acid. It has been shown that the bio-produced COSs with a wide range of Mwt and DP can provide a promising antioxidant activity as close to that of ascorbic acid in in vitro experiments. This result is consistent with El-Sayed et al. ^30^ and Doan et al. ^36^, who found that COSs at 1000 µg/mL exhibited the highest antioxidant activity, with DPPH scavenging activity of 86.97% and a dose-dependent increase, reaching its maximum at 1200 µg/mL. The IC_50_ value of COSs samples was 55.55 ± 1.49 µg/mL. This is in contrast to Laokuldilok et al. ^37^., who reported that the IC_50_ value of their tested COSs was 2710 µg/ml.

According to a previous report, COSs possess specific antioxidant activities and can rejuvenate various enzymes (e.g., catalase, SOD, GSH, and GPx) impaired by oxidative stressors ^10^. In this study, the activity of the enzymes studied (Catalase, SOD, GSH, and GPx) increased in stomach, liver, and serum samples in the HFD group with 0.5% COSs compared with the normal diet and HFD groups (*p* < 0.0001). These results agree with the studies by Qu and Han ^22^ and Yu et al. ^38^, which reported elevated levels of CAT, GPx, and SOD in the liver, serum, and stomach of mice upon treatment with HFD and 0.5% COSs. Wu et al. ^7^ study also found that COSs increased SOD and GSH levels in mice’s blood samples (*p* < 0.01). Notably, COSs resulted in the highest GPx levels, indicating strong stimulation of enzymatic antioxidant defense and improved systemic antioxidant defense.

The antibiofilm activity of COSs was reported against biofilm-forming *S. aureus* and *P. aeruginosa* bacteria. It has been noted that COSs at a concentration of 62.5 µg/mL could efficiently inhibit biofilm formed by *S. aureus*, while a higher concentration of COSs (125 µg/mL) was enough to inhibit biofilm formation of *P. aeruginosa*. The higher resistance of *P. aeruginosa* may be attributed to its more complex biofilm structure and stronger defense mechanisms. These results agree with the study by Khan et al. ^13^, which reported that a low concentration of the COSs studied was significantly effective at inhibiting S. aureus biofilm formation (< 100 µg/mL). Therefore, the efficacy of COSs as anti-biofilm agents for treating pseudomonal biofilm-associated infections is promising. It is recommended that the antibiofilm activities of COSs can be enhanced by combining the COSs products with other antibiotics, such as Streptomycin ^15^.

The crystal violet assay demonstrated a significant reduction in surface-associated biofilm biomass following treatment. However, this colorimetric method measures the total attached biomass, including bacterial cells and components of the extracellular biofilm matrix, and does not provide direct information about the mechanism responsible for biofilm inhibition. Therefore, the observed reduction may have resulted from interference with initial bacterial adhesion, inhibition of biofilm maturation, disruption of extracellular polymeric substances, enhanced detachment of established biofilms, reduced bacterial viability, or a combination of these effects. Although membrane disruption and inhibition of adhesion have been proposed as possible mechanisms for similar antimicrobial agents, these mechanisms were not directly investigated in the present study and should not be considered experimentally confirmed ^39, 40^.

A limitation of the present study is that antibiofilm activity was evaluated solely by the crystal violet assay. Although this assay is widely used to quantify changes in total attached biofilm biomass, it cannot differentiate between effects on bacterial viability, initial adhesion, biofilm maturation, extracellular matrix production, or biofilm dispersal. Consequently, the underlying mechanism of the observed antibiofilm activity remains to be elucidated through complementary mechanistic and microscopic analyses.

## Conclusions

In conclusion, the COSs obtained in this study showed potential antitumor activity against breast, liver, colorectal, and lung cancers. Also, they exhibit potential antioxidant activity in both in vivo and in vitro studies, as well as antibiofilm activity against biofilm-forming S. aureus and P. aeruginosa.

This study primarily contributes to the development of novel, safe, and multifunctional bioactive COSs as compounds for biomedical applications. The bio-produced COSs provide significant therapeutic potential due to their combined antitumor, antioxidant, and antibiofilm properties. Their antitumor activity supports their use as promising candidates for cancer therapy, either alone or as adjuvants to enhance efficacy and reduce toxicity of conventional treatments. The antioxidant capacity of COSs helps mitigate oxidative stress, a key factor in the pathogenesis of many chronic diseases, thereby broadening their preventive and therapeutic relevance. Additionally, their antibiofilm activity addresses a critical challenge in clinical microbiology, as biofilm-associated infections are highly antibiotic-resistant and often persist. Further studies using Annexin V/PI flow cytometry, caspase activity assays, mitochondrial membrane potential analysis, and apoptosis-related protein expression analyses are required to clarify the underlying mechanism. Other studies using membrane-integrity assays, viable cell counting, microscopic biofilm analysis, extracellular polymeric substance quantification, adhesion assays, and expression analysis of biofilm-associated genes are required to determine the predominant mechanism.

## Data Availability

All data in this article were gathered, prepared, and analyzed during this study. All other data are available.

## Abbreviations

ANOVA: Two-way analysis of variance
COSs: Chitooligosaccharides
DD: Degree of deacetylation
DP: Degree of polymerization
DPPH: 2,2-diphenyl-1-picrylhydrazyl
GPx: Glutathione peroxidase
GSH: Reduced glutathione
HFD: High Fat Diet
IC_50_: Inhibitory concentration
RCMV: Regional Center for Mycology & Biotechnology
SOD: Superoxide dismutase
